# TBGA: a large-scale Gene-Disease Association dataset for Biomedical Relation Extraction

**DOI:** 10.1186/s12859-022-04646-6

**Published:** 2022-03-31

**Authors:** Stefano Marchesin, Gianmaria Silvello

**Affiliations:** Department of Information Engineering, University of Padova, Padova, Italy

**Keywords:** Weak supervision, Biomedical Relation Extraction, Gene-Disease Association

## Abstract

**Background:**

Databases are fundamental to advance biomedical science. However, most of them are populated and updated with a great deal of human effort. Biomedical Relation Extraction (BioRE) aims to shift this burden to machines. Among its different applications, the discovery of Gene-Disease Associations (GDAs) is one of BioRE most relevant tasks. Nevertheless, few resources have been developed to train models for GDA extraction. Besides, these resources are all limited in size—preventing models from scaling effectively to large amounts of data.

**Results:**

To overcome this limitation, we have exploited the DisGeNET database to build a large-scale, semi-automatically annotated dataset for GDA extraction. DisGeNET stores one of the largest available collections of genes and variants involved in human diseases. Relying on DisGeNET, we developed TBGA: a GDA extraction dataset generated from more than 700K publications that consists of over 200K instances and 100K gene-disease pairs. Each instance consists of the sentence from which the GDA was extracted, the corresponding GDA, and the information about the gene-disease pair.

**Conclusions:**

TBGA is amongst the largest datasets for GDA extraction. We have evaluated state-of-the-art models for GDA extraction on TBGA, showing that it is a challenging and well-suited dataset for the task. We made the dataset publicly available to foster the development of state-of-the-art BioRE models for GDA extraction.

**Supplementary Information:**

The online version contains supplementary material available at 10.1186/s12859-022-04646-6.

## Background

Curated databases, such as UniProt [1], DrugBank [2], CTD [3], IUPHAR/BPS [4], Reactome [5], OMIM [6], or COSMIC [7], are pivotal to the development of biomedical science. Such databases are usually populated and updated with expensive and time-consuming human effort [8], that slows down the biological knowledge discovery process. To overcome this limitation, Biomedical Information Extraction (BioIE) aims to shift population and curation processes to machines by developing effective computational tools that automatically extract meaningful facts from the vast unstructured scientific literature [9, 10]. Once extracted, machine-readable facts can be fed to downstream tasks to ease biological knowledge discovery. Among the various tasks, the discovery of Gene-Disease Associations (GDAs) is one of the most pressing challenges to advance precision medicine and drug discovery [11], as it helps to understand the genetic causes of diseases [12]. Thus, the automatic extraction and curation of GDAs is key to advance precision medicine research and provide knowledge to assist disease diagnostics, drug discovery, and therapeutic decision-making.

Most datasets for GDA extraction are hand-labeled corpora [13–15]. Among them, EU-ADR [13] only contains a small portion of GDA instances, making it difficult to train robust RE models for GDA extraction. On the other hand, PolySearch [14] only focuses on ten specific diseases, which are not sufficient to develop comprehensive models. Similarly, CoMAGC [15] only comprises gene-cancer associations on prostate, breast, and ovarian cancers. Hence, all datasets lack enough GDA heterogeneity to train effective RE models. Furthermore, hand-labeling data is an expensive process requiring large amounts of time to expert biologists and, therefore, all of these datasets are limited in size.

To address this limitation, distant supervision has been proposed [16]. Under the distant supervision paradigm, all the sentences mentioning the same pair of entities are labeled by the corresponding relation stored within a source database. The assumption is that if two entities participate in a relation, at least one sentence mentioning them conveys that relation. As a consequence, distant supervision generates a large number of false positives, since not all sentences express the relation between the considered entities. To counter false positives, the RE task under distant supervision can be modeled as a Multi-Instance Learning (MIL) problem [17–20]. With MIL, the sentences containing two entities connected by a given relation are collected into bags labeled with such relation. Grouping sentences into bags reduces noise, as a bag of sentences is more likely to express a relation than a single sentence. Thus, distant supervision alleviates manual annotation efforts, and MIL increases the robustness of RE models to noise.

Since the advent of distant supervision, several datasets for RE have been developed under this paradigm for news and web domains [16, 18, 21, 22], and recently also for biomedical science [10, 23, 24]. The most relevant biomedical datasets are BioRel [24]—a large-scale dataset for domain-general Biomedical Relation Extraction (BioRE)—and DTI [10]—a large-scale dataset developed to extract Drug–Target Interactions (DTIs). However, despite the success of distant supervision for RE tasks, its evaluation is known to be flawed [25, 26]. In this regard, previous works either employ inconsistent and expensive approaches to manually evaluate a small sample of model predictions or test models directly on distant-labeled data—which are inherently noisy and can skew the model’s performance. Only recently some progress has been made towards enhancing distantly-supervised datasets with human annotations [25–28].

Regarding GDA datasets, Bravo et al. [27] developed a semi-automatically annotated corpus based on the (GAD) [29], a retired archive of human genetic association studies of complex diseases. GAD provides the sentence in which a GDA is stated, but omits the information on the exact location of the gene and the disease within such sentence. Thus, the authors were required to perform Named Entity Recognition (NER)—which inevitably introduces noise into the annotation pipeline—to identify genes and diseases within GAD sentences. Once identified, the authors kept those sentences where the gene and disease reflect a GDA annotated by GAD curators as positive or negative. Then, to store false GDAs—that is, GDAs where the gene and the disease co-occur within a sentence but are not semantically associated—Bravo et al. selected sentences with co-occurring genes and diseases that were not annotated by GAD curators as GDAs. Similarly, Nourani and Reshadat [28] exploited DisGeNET [12] to develop a semi-automatically annotated dataset for GDA extraction. DisGeNET is one of the largest available collections of genes and variants involved in human diseases, integrating data from expert-curated repositories, Genome-Wide Association Studies (GWAS) catalogs [30], animal models, and scientific literature. For each GDA, DisGeNET provides the publication(s) supporting the association, a representative sentence from each publication, the original source, as well as information on the gene and disease involved in the association. Hence, the authors kept the GDAs—and the corresponding sentences—coming from DisGeNET curated resources as true instances, whereas they obtained false GDAs through distant supervision by selecting sentences where co-occurring genes and diseases do not participate in any GDA within DisGeNET. However, despite the use of large source databases and distant supervision, both the produced datasets are limited in size and have not been designed for a MIL setting, which is the de facto standard for distantly-supervised datasets.

To overcome the limited size of current manually or semi-automatically annotated GDA datasets, as well as the noisy nature of fully distantly-supervised BioRE datasets, we make the following contributions. First, we present TBGA, a novel large-scale, semi-automatically annotated dataset for GDA extraction based on DisGeNET. We chose DisGeNET as source database since it is one of the most comprehensive databases for GDAs [31], integrating several expert-curated resources, such as UniProt [1], CTD [3], and PsyGeNET [32]. Furthermore, DisGeNET spans several different types of GDAs, as opposed to other databases like OMIM [6], COSMIC [7], TTD [33], BioMuta and BioXpress [34], which only focus on specific GDA types. Specifically, we used the portion of DisGeNET with curated resources to make validation and test sets, whereas we used the rest for training. On the other hand, we generated false GDAs by selecting sentences where co-occurring genes and diseases do not participate in DisGeNET GDAs. Compared to the dataset developed by Bravo et al. [27], TBGA exploits DisGeNET—which is three orders of magnitude larger than GAD—to gather true GDAs as well as to generate false ones. Regarding the dataset by Nourani and Reshadat [28], TBGA fully exploits DisGeNET resources and does not limit to curated ones. In this way, all the available expert-curated resources can be used to build validation and test sets, making the produced dataset larger than previous attempts and more realistic than fully distantly-supervised datasets. As a side note, we do not compare TBGA to the fully distantly-supervised GDA dataset by Teng et al. [23] as the dataset is not publicly available. To the best of our knowledge, TBGA is the largest available dataset for GDA extraction.

Secondly, we trained and tested several state-of-the-art RE models on TBGA to create a large and realistic benchmark for GDA extraction. We built models using OpenNRE [35], an open and extensible toolkit for Neural Relation Extraction (NRE). The choice of OpenNRE eases the re-use of the dataset and the models developed for this work to future researchers.

Finally, we publicly release TBGA on Zenodo [36], whereas we store source code and scripts to train and test RE models in a publicly available GitHub repository [37]. Besides, thanks to the continuous growth of DisGeNET, the released dataset can be updated and expanded regularly.

## Results

TBGA is the first large-scale, semi-automatically annotated dataset for GDA extraction. The dataset consists of three text files, corresponding to train, validation, and test sets, plus an additional JSON file containing the mapping between relation names and IDs. Each record in train, validation, or test files corresponds to a single GDA extracted from a sentence, and it is represented as a JSON object with the following attributes:

- text: sentence from which the GDA was extracted.
- relation: relation name associated with the given GDA.
- h: JSON object representing the gene entity, composed of:
  - id: NCBI Entrez ID associated with the gene entity.
  - name: NCBI official gene symbol associated with the gene entity.
  - pos: list consisting of starting position and length of the gene mention within text.
- t: JSON object representing the disease entity, composed of:
  - id: UMLS Concept Unique Identifier (CUI) associated with the disease entity.
  - name: UMLS preferred term associated with the disease entity.
  - pos: list consisting of starting position and length of the disease mention within text.

If a sentence contains multiple gene-disease pairs, the corresponding GDAs are split into separate data records.

**Fig. 1:**
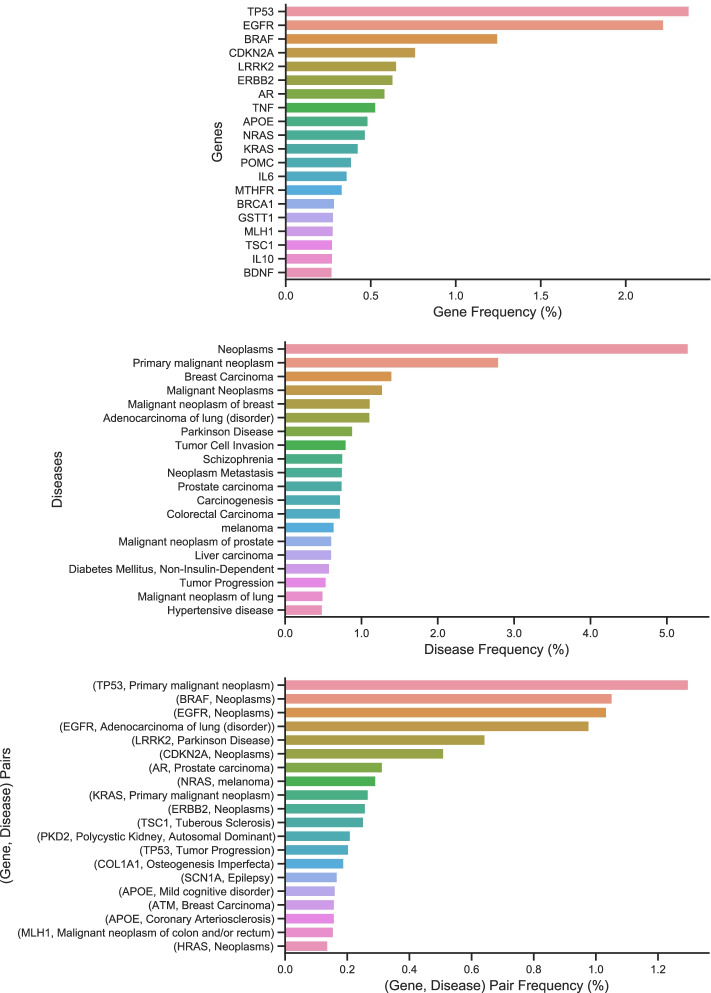
The 20 most frequent genes, diseases, and GDAs within TBGA

Overall, TBGA contains over 200,000 instances and 100,000 bags. Table 1 reports per-relation statistics for the dataset. Notice the large number of Not Associated (NA) instances. Moreover, Fig. 1 depicts the 20 most frequent genes, diseases, and GDAs within TBGA. The most frequent genes are tumor suppressor genes, such as TP53 and CDKN2A, and (proto-)oncogenes, like EGFR and BRAF. Among the most frequent diseases, we have neoplasms such as breast carcinoma, lung adenocarcinoma, and prostate carcinoma. As a consequence, the most frequent GDAs are gene-cancer associations.

**Table 1:** Per-relation statistics for TBGA

Granularity	Split	Therapeutic	Biomarker	Genomic alterations	NA
Sentence-level	Train	3139	20,145	32,831	122,149
	Validation	402	2279	2306	15,206
	Test	384	2315	2209	15,608
Bag-level	Train	2218	13,372	12,759	56,698
	Validation	331	2019	1147	6994
	Test	308	2068	1122	6996

Statistics are reported separately for each data split. Columns represent, from left to right, the considered granularity level, the data split, and the number of instances and bags associated with Therapeutic, Biomarker, Genomic Alterations, and NA relations

TBGA is two orders of magnitude larger than current available datasets for GDA extraction [13–15, 27, 28]. Moreover, TBGA focuses on different association types, whereas most of current datasets only consider positive, negative, or false GDAs. The only exception is CoMAGC [15], where relations focus on different aspects of the gene expression changes and their association with cancer. Therefore, training and then testing RE models on TBGA allows for a more fine-grained and realistic evaluation that helps building effective solutions for GDA extraction. Table 2 compares global statistics between TBGA, EU-ADR [13], CoMAGC [15], PolySearch [14], GAD [27], and GDAE [28] datasets.

**Table 2:** Global statistics comparison between TBGA, EU-ADR [13], CoMAGC [15], PolySearch [14], GAD [27], and GDAE [28] datasets

Dataset	Annotation	Instances	Publications	Inst.s/pub.	Genes	Diseases	Relations
CoMAGC	Manual	821	408	2.01	538	3	15
EU-ADR	Manual	355	65	5.46	221	118	4
PolySearch	Manual	522	374	1.40	245	10	2
GAD	Weak	5329	4112	1.30	1139	535	3
GDAE	Weak	8000	5875	1.36	3635	1904	2
TBGA	Weak	218,973	134,059	1.63	11,784	9199	4

Columns represent, from left to right, the considered dataset, the type of annotation, the total number of instances and publications, the average number of instances per publication, as well as the total number of genes, diseases, and relations

On the other hand, compared to current large-scale, fully distantly-supervised BioRE datasets—i.e., BioRel [24] and DTI [10]—TBGA contains expert-curated data. Hence, TBGA represents a more accurate benchmark than fully distantly-supervised datasets where to train and test RE models—helping to understand the current status and future steps required to improve BioRE research [26]. Despite the use of expert-curated data, TBGA has a size comparable to that of fully distantly-supervised BioRE datasets. Besides, with the continuous growth of DisGeNET, the size of TBGA can further increase. Table 3 compares global statistics between TBGA, DTI [10], and BioRel [24] datasets.

**Table 3:** Global statistics comparison between TBGA, BioRel [24], and DTI [10] datasets

Dataset	Split	Instances	Bags	Inst.s/bag	Relations
BioRel	Train	534,277	39,969	13.37	125
	Validation	114,506	20,675	5.54	
	Test	114,565	20,756	5.52	
DTI	Train	604,303	472,033	1.28	6
	Validation	6133	4769	1.29	
	Test	6312	4817	1.31	
TBGA	Train	178,264	85,047	2.10	4
	Validation	20,193	10,491	1.92	
	Test	20,516	10,494	1.96	

Statistics are reported separately for each data split. Columns represent, from left to right, the considered granularity level, the data split, the total number of instances and bags, the average number of instances per bag, as well as the total number of relations

## Discussion

### **Data validation**

In order to validate TBGA, we conducted comprehensive experiments with state-of-the-art RE models under the Multi-Instance Learning (MIL) setting. MIL is the typical setting used for distantly-supervised RE, where sentences are divided into bags based on pairs of entities and the prediction of relations occurs at bag-level. For example, the following two instances compose the “ADM-Schizophrenia” bag, where the target relation is Biomarker. **Instance 1:** “Our data support that **ADM** may be associated with the pathophysiology of **schizophrenia**, although the cause of the association needs further study.” **Instance 2:** “These findings suggest the possible role of **ADM** and SEPX1 as biomarkers of **schizophrenia**.”

Below, we first describe the experimental setup and then present the results.

#### Experimental setup

##### Datasets

We performed experiments on three different datasets: TBGA, DTI, and BioRel. We used TBGA as a benchmark to evaluate RE models for GDA extraction under the MIL setting. On the other hand, we used DTI and BioRel only to validate the soundness of our implementation of the baseline models.

##### Evaluation measures

We evaluated RE models using the Area Under the Precision-Recall Curve (AUPRC). AUPRC is a popular measure to evaluate distantly-supervised RE models, which has been adopted by OpenNRE [35] and used in several works, such as [10, 24]. For experiments on TBGA, we also computed Precision at k items (P@k) and plotted the precision-recall curves.

##### Aggregation strategies

We adopted two different sentence aggregation strategies to use RE models under the MIL setting: average-based (AVE) and attention-based (ATT) [38]. The average-based aggregation assumes that all sentences within the same bag contribute equally to the bag-level representation. In other words, the bag representation is the average of all its sentence representations. On the other hand, the attention-based aggregation represents each bag as a weighted sum of its sentence representations, where the attention weights are dynamically adjusted for each sentence.

##### Baseline models

We considered the main state-of-the-art RE models to perform experiments: CNN [39], PCNN [40], BiGRU [10, 24, 41], BiGRU-ATT [10, 42], and BERE [10]. A detailed description of these RE models, along with information on parameter settings and hyper-parameter tuning, can be found in Additional file 1.

#### Experimental results

We report the results for two different experiments. The first experiment aims to validate the soundness of the implementation of the considered RE models. To this end, we trained and tested the RE models on DTI and BioRel datasets, and we compared the AUPRC scores we obtained against those reported in the original works [10, 24]. For this experiment, we only compared the RE models and aggregation strategies that were used in the original works. The results and discussion of the experiment can be found in Additional file 2. The second experiment uses TBGA as a benchmark to evaluate RE models for GDA extraction. In this case, we trained and tested all the considered RE models using both aggregation strategies. For each RE model, we reported the AUPRC and P@k scores, and we plotted the precision-recall curve.

##### GDA benchmarking

**Fig. 2:**
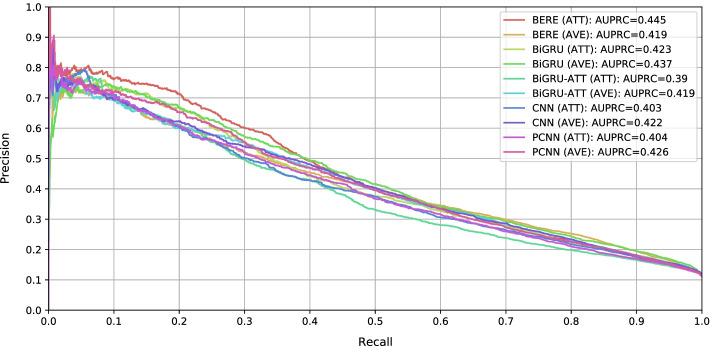
Precision-Recall curves for RE models on TBGA dataset. RE models are evaluated using both aggregation strategies—that is, average-based (AVE) and attention-based (ATT). Therefore, precision-recall curves are plot for each aggregation strategy

Table 4 shows the AUPRC and P@k scores of RE models on TBGA, whereas Fig. 2 plots the corresponding precision-recall curves. Given the RE models performance and precision-recall curves, we make the following observations.

1. The performances achieved by RE models on TBGA indicate a high complexity of the GDA extraction task. When recall is smaller than 0.1, all RE models have precision greater than 0.7. However, at higher recall values, models performance decrease sharply. In particular, when recall is greater than 0.4, no RE model achieves precision values greater than or equal to 0.5. The task complexity is further supported by the lower performances obtained by top-performing RE models on TBGA compared to DTI and BioRel (cf. Additional file 2: Table S2).
2. CNN, PCNN, BiGRU, and BiGRU-ATT RE models behave similarly. Among them, BiGRU-ATT has the worst performance. This suggests that replacing BiGRU max pooling layer with an attention layer proves less effective. Overall, the best AUPRC and P@k scores are achieved by BERE when using the attention-based aggregation strategy. This highlights the effectiveness of fully exploiting sentence information from both semantic and syntactic aspects [10]. BERE top performance can also be observed by looking at its precision-recall curve, which remains constantly above the other curves up to recall 0.4, where it stabilizes with the others. Nevertheless, most of RE models—regardless of the considered aggregation strategy—show precision drops at early recall values, not greater than 0.4.
3. In terms of AUPRC, the attention-based aggregation proves less effective than the average-based one. On the other hand, attention-based aggregation provides mixed results on P@k measures. Although in contrast with the results obtained in general-domain RE [38], this trend is in line with the results found by Xing et al. [24] on BioRel, where RE models using an average-based aggregation strategy achieve performance comparable to or higher than those using an attention-based one. The only exception is BERE, whose performance using the attention-based aggregation outperforms the one using the average-based strategy.

Thus, the obtained results suggest that TBGA is a challenging dataset for GDA extraction and, in general, for BioRE.

**Table 4:** RE models performance on TBGA dataset

Model	Strategy	AUPRC	P@50	P@100	P@250	P@500	P@1000
CNN	AVE	0.422	**0.780**	0.760	0.744	0.696	0.625
	ATT	0.403	**0.780**	0.760	0.788	0.710	0.624
PCNN	AVE	0.426	**0.780**	**0.780**	0.744	0.720	0.664
	ATT	0.404	0.760	0.750	0.744	0.700	0.628
BiGRU	AVE	0.437	0.620	0.720	0.724	0.730	0.678
	ATT	0.423	0.760	0.750	0.748	0.726	0.666
BiGRU-ATT	AVE	0.419	0.740	0.740	0.748	0.694	0.615
	ATT	0.390	0.680	0.760	0.756	0.702	0.631
BERE	AVE	0.419	0.700	0.710	0.720	0.704	0.620
	ATT	**0.445**	**0.780**	**0.780**	**0.800**	**0.764**	**0.709**

Columns represent, from left to right, the considered RE model, the aggregation strategy, the AUPRC score, as well as the P@50, P@100, P@250, P@500, and P@1000 scores. For each measure, bold values represent the best scores

### **Re-use potential**

TBGA complies with the format required by OpenNRE [35] to train and test RE models. We chose to structure the dataset in this way to ease its re-use to future researchers. OpenNRE already provides several RE models that can be used directly on TBGA. In addition, we have also used OpenNRE to implement widely-used missing RE models.

We used TBGA as a benchmark to evaluate RE models under the MIL setting—which is the typical setting for the RE task under distant supervision. In other words, we trained and tested RE models at bag-level. However, TBGA contains sentence-level expert-curated annotations in validation and test sets. Thus, researchers can also use TBGA to train RE models at bag-level and evaluate them on sentence-level expert-curated data—which is an emerging setting for distantly-supervised, manually enhanced datasets [25, 26]. To this end, no format changes are required to make TBGA compliant with the alternative setting.

## Conclusions

We have presented a large-scale, semi-automatically annotated dataset for Gene-Disease Association (GDA) extraction. Automatic GDA extraction is one of the most relevant tasks of BioRE. We have used TBGA as a benchmark to evaluate state-of-the-art BioRE models on GDA extraction. The results suggest that TBGA is a challenging dataset for this task. Besides, the large size of TBGA—along with the presence of expert-curated annotations in its validation and test sets—makes it more realistic than fully distantly-supervised BioRE datasets.

## Methods

**Fig. 3:**
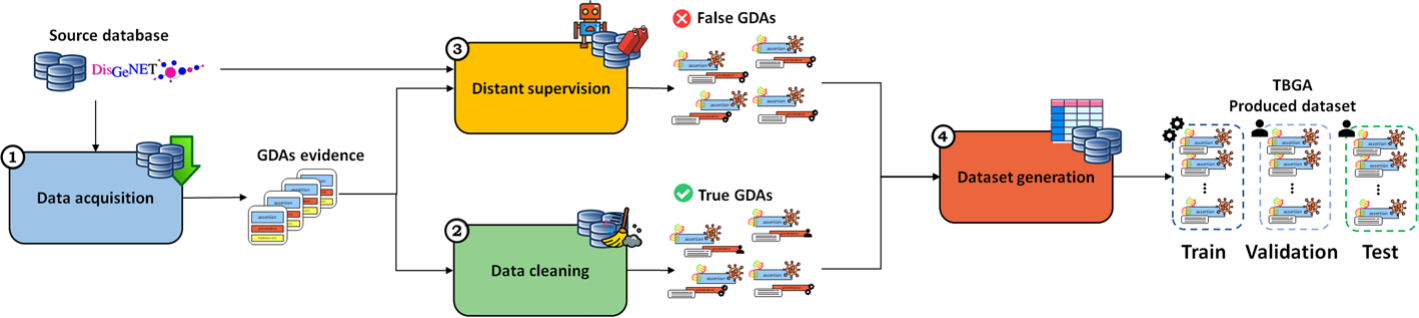
Overview of the TBGA creation process. The process consists of four steps: (1) data acquisition; (2) data cleaning; (3) distant supervision; and (4) dataset generation

The process to create TBGA consisted of four steps: data acquisition, data cleaning, distant supervision, and dataset generation. Figure 3 illustrates the overall procedure.

### **Data acquisition**

**Fig. 4:**
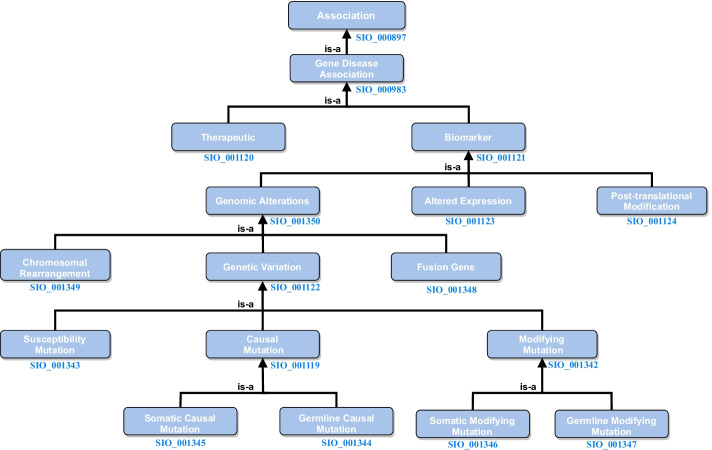
DisGeNET association type ontology. For each association type, we also report its SIO identifier

The data used to generate TBGA comes from DisGeNET [12]. DisGeNET collects data on genotype-phenotype relationships from several resources and covers most of human diseases, including Mendelian, complex, environmental and rare diseases, as well as disease-related traits. According to the type of resource, DisGeNET organizes gene-disease data into one of four categories: Curated, Animal Models, Inferred, and Literature. Curated data contains GDA provided by expert-curated resources; Animal Models data includes GDA from resources containing information about rat and mouse models of disease; Inferred data refers to GDAs inferred from the Human Phenotype Ontology (HPO) [43] and from Variant-Disease Associations (VDAs); and Literature data provides GDAs extracted from the scientific literature using text-mining techniques [27, 44, 45]. For a seamless integration of such GDAs, DisGeNET classifies them by different association types, which are defined in the DisGeNET association type ontology. A detailed description of each association type can be found on the DisGeNET platform [46]. Figure 4 depicts the DisGeNET association type ontology, where we also report the Semanticscience Integrated Ontology (SIO) [47] identifiers of the different association types.

We acquired data from DisGeNET v7.0 to build TBGA. This version of DisGeNET contains 1,134,942 GDAs, involving 21,671 genes and 30,170 diseases, disorders, traits, and clinical or abnormal human phenotypes. We accessed DisGeNET data through the web interface [46], where we used the Browse functionality to retrieve GDAs along with supporting evidence. We gathered data from all four resource categories. Moreover, we filtered out data with no PubMed IDentifier (PMID) to avoid retrieving GDAs without a sentence supporting the association.

### **Data cleaning**

The data acquired from DisGeNET underwent a data cleaning process. First, we filtered data based on the presence of tags surrounding the gene and disease mentions within sentences. In other words, we restricted to GDAs having representative sentences where the gene and the disease are highlighted. Then, we stripped gene and disease tags from text and we stored the exact location of gene and disease mentions within sentences. Since DisGeNET integrates data from various resources, there might be duplicate evidence for the same GDA. In this case, we discarded duplicates and prioritized data coming from expert-curated resources.

From each instance resulting from the data cleaning process, we considered the following attributes: the original source, the publication supporting the association, the representative sentence, the association type, as well as information on the gene and disease involved in the association. Regarding genes, we kept the NCBI Entrez [48] identifiers, the NCBI official gene symbols, and the gene locations within sentences. As for diseases, we stored the UMLS [49] CUIs, the UMLS preferred terms, and the disease locations in text.

### **Distant supervision**

To effectively train RE models, false GDAs are also required—i.e., instances where co-occurring genes and diseases are not semantically associated. However, DisGeNET stores only true GDAs. To overcome this limitation, we used distant supervision [16] to obtain false GDAs from the sentences contained within the abstract or title of the PubMed articles that support the GDAs retrieved in the data acquisition process. To this end, we relied on the 3.6.2rc6 version of MetaMapLite [50], a near real-time NER tool that identifies UMLS concepts within biomedical text. MetaMapLite returns, among other information, the CUI, the preferred term, and the location in text of the identified UMLS concepts. Thus, we used MetaMapLite to identify gene and disease UMLS concepts within sentences. For each identified concept, we stored its CUI, preferred term, and location in text. Then, we performed the following steps to generate false GDAs.

1. We restricted to sentences where the co-occurring genes and diseases come from DisGeNET. The search for false GDAs among the genes and diseases of DisGeNET aimed to reduce false negatives and to obtain gene-disease pairs that were more likely not to be semantically associated.
2. We filtered out instances where gene mentions matched common words. For instance, when all letters are in uppercase, the words FOR and TYPE are, by convention [51], aliases for the WWOX and SGCG genes. Therefore, when the gene mentions identified by MetaMapLite matched such (and other) common words, we kept the corresponding instances only if the matched words were in uppercase. As common words, we considered the set of most frequent words provided by Peter Norvig [52], which were derived from the Google Web Trillion Word Corpus [53].
3. We used the 2020AA UMLS MRCONSO file [54] to build a disease dictionary that stored UMLS preferred terms, lexical variants, alternate forms, short forms, and synonyms of the DisGeNET diseases. The MRCONSO file contains one row for each occurrence of each unique string or concept name within each source vocabulary of the UMLS Metathesaurus. Thus, we only kept instances where disease mentions exact-matched dictionary terms. In this way, we removed partial matches identified by MetaMapLite and, as a consequence, we reduced erroneous disease mentions.
4. Of the remaining instances, we only took those whose gene-disease pairs did not belong to any GDA within DisGeNET and we labeled them as NA.

For each instance generated through distant supervision, we kept the following attributes: the publication and sentence from which the false GDA has been extracted, the NA association type, and information on the co-occurring gene and disease. For genes, we first mapped UMLS CUIs to NCBI Entrez IDs, and then we stored them together with NCBI official gene symbols and gene locations in text. On the other hand, for diseases, we stored UMLS CUIs, UMLS preferred terms, as well as disease locations in text.

### **Dataset generation**

The sets of true and false instances obtained from the data cleaning and distant supervision processes were used to generate TBGA. We considered different associations from the DisGeNET association type ontology to build the dataset. Specifically, we adopted the Therapeutic, Biomarker, and Genomic Alterations associations types as relations. Instead, we did not consider the Altered Expression and Post-translational Modification association types—although at the same level of Genomic Alterations—as we lacked curated data for them. In addition to true associations, we also considered the false association NA.

The steps required to create TBGA were the following:

1. We performed a normalization process to convert DisGeNET association types to TBGA relations. In this regard, given the hierarchical structure of the DisGeNET association type ontology, we could normalize finer association types to their coarser ancestors. For instance, a Genetic Variation association is also a Genomic Alterations one, which, in turn, is a Biomarker association (cf. Fig. 4). Thus, we mapped association types finer than Genomic Alterations to Genomic Alterations itself. On the other hand, instances involving the same gene-disease pair from the same sentence can have Biomarker or Genomic Alterations association types depending on the considered resource. This situation occurs because instances are generated by different biologists or using different text-mining techniques. In these cases, we removed the instances associated with Biomarker to keep gene-disease pairs associated with Genomic Alterations, which represents a finer—and thus more precise—association type than Biomarker.
2. We divided true instances among training, validation, and test sets based on the resource category. We used Curated data for validation and test, whereas Animal Models, Inferred, and Literature data for training. The only exception was Therapeutic, where we lacked enough data for training. In this case, we also used Curated data for training, setting an 80/10/10 ratio among training, validation, and test sets.
3. We balanced the number of true instances among the dataset relations. For Biomarker and Genomic Alterations, we split Curated data evenly between validation and test. Then, we kept the same ratio that exists among relations in validation and test sets also in training. Since we model the BioRE task as a MIL problem, we downsampled over-represented relations—i.e., Biomarker and Genomic Alterations—at the bag-level rather than at the sentence-level to obtain the desired ratio among relations.
4. We want TBGA to reflect the sparseness of GDAs in biomedical literature. Assuming we randomly sample gene and disease mentions from a sentence of a given scientific article, it is very likely that no association occurs between them. Therefore, similar to previous works [10, 24], we included a large number of false instances into training, validation, and test sets to make TBGA sparse. For each set, we sampled a number of NA bags twice the number of bags associated with true relations.
5. We removed from the training set the bags whose gene-disease pairs also belong to validation and test sets. This operation avoids to introduce bias at inference time, as RE models cannot exploit training knowledge on the gene-disease pair.

We provide statistics regarding the different steps of data cleaning and dataset generation for true instances in Table 5. As for NA statistics, we performed distant supervision on more than 700,000 publications, obtaining 152,963 instances and 70,688 bags—which are associated with 83,501 publications and involve 9167 different genes and 5151 different diseases.

**Table 5:** Global and per-relation statistics for data cleaning and dataset generation

Granularity	Target	Raw	Data cleaning		Dataset generation	
			TS	DR	RN	DB
Global	Publications	707,390	572,981	572,607	447,280	57,675
	Genes	21,118	17,658	17,658	17,658	8827
	Diseases	23,433	17,032	17,023	17,023	6964
Therapeutic	Instances	10,744	4132	3925	3925	3925
	Bags	6872	2939	2857	2857	2,857
Biomarker	Instances	1,530,072	1,080,089	1,075,327	580,053	24,739
	Bags	605,826	460,334	460,276	383,358	17,459
Genomic Alterations	Instances	849,472	531,601	516,630	516,630	37,346
	Bags	289,693	202,548	202,045	202,045	15,028

Columns represent, from left to right, the considered granularity level, the target item, the raw (initial) statistics, and the statistics after each Data Cleaning and Dataset Generation step. The steps are: TS, DR, RN, and DB

## Supplementary Information

**Additional file 1.** BioRE models description and settings. Detailed description of the considered RE models, along with information on parameter settings andhyper-parameter tuning.

**Additional file 2.** Baselines validation. Results and discussion of the experiment performed to validate the soundness of the implementation of theconsidered RE models.

## Data Availability

The TBGA dataset is publicly available on Zenodo [36]. On the other hand, the scripts used to compute global and per-relation dataset statistics, convert DTI and BioRel formats to OpenNRE, train and test RE models, as well as the source code to implement widely-used RE models not available in OpenNRE, are publicly available on GitHub [37]. All the underlying libraries used in this work are open-source. The complete list of libraries and their versions are reported in the GitHub repository.

## Abbreviations

AUPRC: Area Under the Precision-Recall Curve
BiGRU: Bidirectional Gated Recurrent Unit Neural Network
BioIE: Biomedical Information Extraction
BioRE: Biomedical Relation Extraction
CNN: Convolutional Neural Network
CUI: Concept Unique Identifier
DB: Data Balancing
DR: Duplicates Removal
DTI: Drug–Target Interaction
ECO: Evidence Ontology
GAD: Genetic Association Database
GDA: Gene-Disease Association
GWAS: Genome-Wide Association Studies
HPO: Human Phenotype Ontology
MIL: Multi-Instance Learning
MeSH: Medical Subject Headings
NA: Not Associated
NER: Named Entity Recognition
NRE: Neural Relation Extraction
PCNN: Piecewise Convolutional Neural Network
PF: Position Feature
PI: Position Indicator
PMID: PubMed IDentifier
P@k: Precision at k items
RDF: Resource Description Framework
RE: Relation Extraction
RN: Relation Normalization
SGD: Stochastic Gradient Descent
SIO: Semanticscience Integrated Ontology
TS: Tag Stripping
VDA: Variant-Disease Association

## References

[1] Bairoch A, Apweiler R (1997). The SWISS-PROT protein sequence data bank and its supplement TrEMBL. Nucleic Acids Res.

[2] Wishart DS, Knox C, Guo A, Shrivastava S, Hassanali M, Stothard P, et al. DrugBank: a comprehensive resource for in silico drug discovery and exploration. Nucleic Acids Res. 2006;34(Database-Issue):668–72. 10.1093/nar/gkj067 PMC1347430 16381955

[3] Mattingly CJ, Colby GT, Forrest JN, Boyer JL (2003). The Comparative Toxicogenomics Database (CTD). Environ Health Perspect..

[4] Harmar AJ, Hills RA, Rosser EM, Jones M, Buneman OP, Dunbar DR, et al. IUPHAR-DB: the IUPHAR database of G protein-coupled receptors and ion channels. Nucleic Acids Res. 2009;37(Database-Issue):680–5. 10.1093/nar/gkn728 PMC2686540 18948278

[5] Joshi-Tope G, Gillespie M, Vastrik I, D’Eustachio P, Schmidt E, de Bono B, et al. Reactome: a knowledgebase of biological pathways. Nucleic Acids Res. 2005;33(Database-Issue):428–32. 10.1093/nar/gki072 PMC540026 15608231

[6] Amberger JS, Bocchini CA, Scott AF, Hamosh A. OMIM.org: leveraging knowledge across phenotype-gene relationships. Nucleic Acids Res. 2019;47(Database-Issue):D1038–43. 10.1093/nar/gky1151 PMC6323937 30445645

[7] Tate JG, Bamford S, Jubb H, Sondka Z, Beare D, Bindal N, et al. COSMIC: the catalogue of somatic mutations in cancer. Nucleic Acids Res. 2019;47(Database-Issue):D941–7. 10.1093/nar/gky1015 PMC6323903 30371878

[8] Buneman P, Cheney J, Tan WC, Vansummeren S. Curated databases. In: Proceedings of the twenty-seventh ACM SIGMOD-SIGACT-SIGART symposium on principles of database systems, PODS 2008, June 9-11, 2008, Vancouver, BC, Canada. ACM; 2008. p. 1–12.

[9] Wang S, Ma J, Yu MK, Zheng F, Huang EW, Han J, et al. Annotating gene sets by mining large literature collections with protein networks, vol. 3–7. Hawaii, USA, January: The Big Island of Hawaii; 2018. p. 601–613. PMC5806628 29218918

[10] Hong L, Lin J, Li S, Wan F, Yang H, Jiang T (2020). A novel machine learning framework for automated biomedical relation extraction from large-scale literature repositories. Nat Mach Intell.

[11] Dugger S, Platt A, Goldstein D (2018). Drug development in the era of precision medicine. Nat Rev Drug Discov.

[12] González JP, Ramírez-Anguita JM, Saüch-Pitarch J, Ronzano F, Centeno E, Sanz F, et al. The DisGeNET knowledge platform for disease genomics: 2019 update. Nucleic Acids Res. 2020;48(Database-Issue):D845–55. 10.1093/nar/gkz1021 PMC7145631 31680165

[13] van Mulligen EM, Fourrier-Réglat A, Gurwitz D, Molokhia M, Nieto A, Trifirò G (2012). The EU-ADR corpus: annotated drugs, diseases, targets, and their relationships. J Biomed Inform.

[14] Cheng D, Knox C, Young N, Stothard P, Damaraju S, Wishart DS. PolySearch: a web-based text mining system for extracting relationships between human diseases, genes, mutations, drugs and metabolites. Nucleic Acids Res. 2008;36(Web-Server-Issue):399–405. 10.1093/nar/gkn296 PMC2447794 18487273

[15] Lee HJ, Shim SH, Song MR, Lee H, Park JC (2013). CoMAGC: a corpus with multi-faceted annotations of gene-cancer relations. BMC Bioinform.

[16] Mintz M, Bills S, Snow R, Jurafsky D (2009). Distant supervision for relation extraction without labeled data.

[17] Dietterich TG, Lathrop RH, Lozano-Pérez T (1997). Solving the multiple instance problem with axis-parallel rectangles. Artif Intell.

[18] Riedel S, Yao L, McCallum A. Modeling relations and their mentions without labeled text. In: Proceedings of machine learning and knowledge discovery in databases, European Conference, ECML PKDD 2010, Barcelona, Spain, September 20–24, 2010. vol. 6323 of LNCS. Springer; 2010. p. 148–163.

[19] Hoffmann R, Zhang C, Ling X, Zettlemoyer LS, Weld DS (2011). Knowledge-based weak supervision for information extraction of overlapping relations.

[20] Surdeanu M, Tibshirani J, Nallapati R, Manning CD. Multi-instance multi-label learning for relation extraction. In: Proceedings of the 2012 joint conference on empirical methods in natural language processing and computational natural language learning, EMNLP-CoNLL 2012, July 12–14, 2012, Jeju Island, Korea. ACL; 2012. p. 455–465.

[21] Han X, Gao T, Lin Y, Peng H, Yang Y, Xiao C, et al. More data, more relations, more context and more openness: a review and outlook for relation extraction. In: Proceedings of the 1st conference of the Asia-Pacific chapter of the association for computational linguistics and the 10th international joint conference on natural language processing, AACL/IJCNLP 2020, Suzhou, China, December 4–7, 2020. ACL; 2020. p. 745–758.

[22] Jat S, Khandelwal S, Talukdar PP. Improving distantly supervised relation extraction using word and entity based attention. In: 6th workshop on automated knowledge base construction, AKBC@NIPS 2017, Long Beach, California, USA, December 8, 2017. OpenReview.net; 2017. p. 1–8.

[23] Teng F, Bai M, Li T. Automatic labeling for gene-disease associations through distant supervision. In: 14th IEEE international conference on intelligent systems and knowledge engineering, ISKE 2019, Dalian, China, November 14–16, 2019. IEEE; 2019. p. 491–497.

[24] Xing R, Luo J, Song T (2020). BioRel: towards large-scale biomedical relation extraction. BMC Bioinform.

[25] Zhu T, Wang H, Yu J, Zhou X, Chen W, Zhang W, et al. Towards accurate and consistent evaluation: a dataset for distantly-supervised relation extraction. In: Proceedings of the 28th international conference on computational linguistics, COLING 2020, Barcelona, Spain (Online), December 8–13, 2020. ICCL; 2020. p. 6436–6447.

[26] Gao T, Han X, Qiu K, Bai Y, Xie Z, Lin Y, et al. Manual evaluation matters: reviewing test protocols of distantly supervised relation extraction. CoRR. 2021. arXiv: abs/2105.09543.

[27] Bravo À, Piñero González J, Queralt-Rosinach N, Rautschka M, Inés Furlong L (2015). Extraction of relations between genes and diseases from text and large-scale data analysis: implications for translational research. BMC Bioinform.

[28] Nourani E, Reshadat V (2020). Association extraction from biomedical literature based on representation and transfer learning. J Theor Biol..

[29] Becker KG, Barnes KC, Bright TJ, Wang SA (2004). The genetic association database. Nat Genet..

[30] Welter D, MacArthur JAL, Morales J, Burdett T, Hall P, Junkins H, et al. The NHGRI GWAS Catalog, a curated resource of SNP-trait associations. Nucleic Acids Res. 2014;42(Database-Issue):1001–6. 10.1093/nar/gkt1229 PMC3965119 24316577

[31] Tanoli Z, Seemab U, Scherer A, Wennerberg K, Tang J, Vähä-Koskela M (2021). Exploration of databases and methods supporting drug repurposing: a comprehensive survey. Brief Bioinform.

[32] Gutiérrez-Sacristán A, Grosdidier S, Valverde O, Torrens M, Bravo À, González JP (2015). PsyGeNET: a knowledge platform on psychiatric disorders and their genes. Bioinformatics.

[33] Li YH, Yu CY, Li XX, Zhang P, Tang J, Yang Q, et al. Therapeutic target database update 2018: enriched resource for facilitating bench-to-clinic research of targeted therapeutics. Nucleic Acids Res. 2018;46(Database-Issue):D1121–7. 10.1093/nar/gkx1076 PMC5753365 29140520

[34] Dingerdissen H, Torcivia-Rodriguez J, Hu Y, Chang TC, Mazumder R, Kahsay RY. BioMuta and BioXpress: mutation and expression knowledgebases for cancer biomarker discovery. Nucleic Acids Res. 2018;46(Database-Issue):D1128–36. 10.1093/nar/gkx907 PMC5753215 30053270

[35] Han X, Gao T, Yao Y, Ye D, Liu Z, Sun M. OpenNRE: an open and extensible toolkit for neural relation extraction. In: Proceedings of the 2019 conference on empirical methods in natural language processing and the 9th international joint conference on natural language processing, EMNLP-IJCNLP 2019, Hong Kong, China, November 3–7, 2019. ACL; 2019. p. 169–174.

[36] Marchesin S, Silvello GTBGA. A large-scale gene-disease association dataset for biomedical relation extraction. Zenodo. 2022. 10.5281/zenodo.5911097. 10.1186/s12859-022-04646-6 PMC8973894 35361129

[37] Marchesin S, Silvello G. GDA extraction. 2022. [Online Accessed 27 Jan 2022]. https://github.com/GDAMining/gda-extraction/.

[38] Lin Y, Shen S, Liu Z, Luan H, Sun M. Neural relation extraction with selective attention over instances. In: Proceedings of the 54th annual meeting of the Association for Computational Linguistics, ACL 2016, August 7–12, 2016, Berlin, Germany, vol. 1: long papers. ACL; 2016. p. 2124–2133.

[39] Zeng D, Liu K, Lai S, Zhou G, Zhao J. Relation classification via convolutional deep neural network. In: Proceedings of COLING 2014, 25th international conference on computational linguistics, technical papers, August 23–29, 2014, Dublin, Ireland. ACL; 2014. p. 2335–2344.

[40] Zeng D, Liu K, Chen Y, Zhao J. Distant supervision for relation extraction via piecewise convolutional neural networks. In: Proceedings of the 2015 conference on empirical methods in natural language processing, EMNLP 2015, Lisbon, Portugal, September 17–21, 2015. ACL; 2015. p. 1753–1762.

[41] Zhang D, Wang D. Relation classification via recurrent neural network. CoRR. 2015. arXiv:abs/1508.01006.

[42] Zhou P, Shi W, Tian J, Qi Z, Li B, Hao H, et al. Attention-based bidirectional long short-term memory networks for relation classification. In: Proceeding of the 54th annual meeting of the Association for Computational Linguistics, ACL 2016, August 7–12, 2016, Berlin, Germany, vol. 2: short papers. ACL; 2016. p. 207–212.

[43] Köhler S, Carmody L, Vasilevsky NA, Jacobsen JOB, Danis D, Gourdine JPF, et al. Expansion of the Human Phenotype Ontology (HPO) knowledge base and resources. Nucleic Acids Res. 2019;47(Database-Issue):D1018–27. 10.1093/nar/gky1105 PMC6324074 30476213

[44] Bundschus M, Dejori M, Stetter M, Tresp V, Kriegel HP (2008). Extraction of semantic biomedical relations from text using conditional random fields. BMC Bioinform.

[45] Bundschus M, Bauer-Mehren A, Tresp V, Furlong LI, Kriegel HP. Digging for knowledge with information extraction: a case study on human gene-disease associations. In: Proceedings of the 19th ACM conference on information and knowledge management, CIKM 2010, Toronto, Ontario, Canada, October 26–30, 2010. ACM; 2010. p. 1845–1848.

[46] DisGeNET Platform; 2010. [Online; Accessed 22 Oct 2021]. https://www.disgenet.org/.

[47] Dumontier M, Baker CJO, Baran J, Callahan A, Chepelev LL, Cruz-Toledo J (2014). The Semanticscience Integrated Ontology (SIO) for biomedical research and knowledge discovery. J Biomed Semant.

[48] Maglott DR, Ostell J, Pruitt KD, Tatusova TA. Entrez gene: gene-centered information at NCBI. Nucleic Acids Res. 2011;39(Database-Issue):52–7. 10.1093/nar/gkq1237 PMC3013746 21115458

[49] Bodenreider O. The Unified Medical Language System (UMLS): integrating biomedical terminology. Nucleic Acids Res. 2004;32(Database-Issue):267–70. 10.1093/nar/gkh061 PMC308795 14681409

[50] Demner-Fushman D, Rogers WJ, Aronson AR (2017). MetaMap Lite: an evaluation of a new Java implementation of MetaMap. J Am Med Inform Assoc.

[51] Bruford EA, Braschi B, Denny P, Jones TEM, Seal RL, Tweedie S (2020). Guidelines for human gene nomenclature. Nat Genet.

[52] Norvig P. Natural language corpus data. In:Segaran T, Hammerbacher J, Editors. Beautiful data. O’Reilly Media, Inc.; 2009. p. 219–242.

[53] Franz A, Brants T. All our N-gram are belong to you; 2006. [Online; Accessed 20 Jan 2022]. http://googleresearch.blogspot.com/2006/08/all-our-n-gram-are-belong-to-you.html.

[54] UMLS MRCONSO: Concept Names and Sources; 2004. [Online; Accessed 20 Jan 2022]. https://www.nlm.nih.gov/research/umls/licensedcontent/umlsarchives04.html.

