# Supplementary material for: TBGA: a large-scale Gene-Disease Association dataset for Biomedical Relation Extraction
Source: BMC Bioinformatics. 2022 Mar 31;23:111. doi: 10.1186/s12859-022-04646-6 (PMC8973894; doi:10.1186/s12859-022-04646-6)
Supplement: Supplementary file 1 — Additional file 1. BioRE models description and settings. Detailed description of the considered RE models, along with information on parameter settings andhyper-parameter tuning. [file 12859_2022_4646_MOESM1_ESM.pdf]

ADDITIONAL FILE 1

TBGA: A Large-Scale Gene-Disease Association Dataset for Biomedical Relation Extraction

Stefano Marchesin* and Gianmaria Silvello

*Correspondence: stefano.marchesin@unipd.it
Department of Information Engineering, University of Padova, Padova, Italy
Full list of author information is available at the end of the article

Experimental setup

Baseline models

We considered the main state-of-the-art Relation Extraction (RE) models to perform experiments.

CNN [1]: the model adopts a max-pooled Convolutional Neural Network (CNN) to extract sentence-level features. In addition to word embeddings, the model also uses Position Features (PFs) to capture structural information – such as the shortest dependency path – between target pairs of entities. Given a word, PFs encode relative word distances between the current word and the pair of entities. PFs are combined with word embeddings and fed into the CNN component. OpenNRE [2] already provides this model under the Multi-Instance Learning (MIL) setting.

PCNN [3]: the model adopts a Piecewise Convolutional Neural Network (PCNN) that first encodes sentences using a CNN and then applies a piecewise max pooling operation. This operation divides each sentence in three segments based on the positions of the two given entities, and returns the maximum value in each segment instead of a single maximum value over the entire sentence. OpenNRE also provides this model under the MIL setting.

BiGRU [4, 5, 6]: the model adopts a max-pooled Bidirectional Gated Recurrent Unit Neural Network (BiGRU) to extract sentence-level features. Instead of PFs, the model uses Position Indicators (PIs) to annotate target entities in each sentence and capture structural information. Compared to PFs, PIs are simpler to use and do not require to change the input representations. We implemented this model in OpenNRE under the MIL setting.

BiGRU-ATT [7, 6]: the model adopts a BiGRU to encode sentences, but replaces the max pooling operation used by Zhang et al. [4] with an attention layer that weighs the contribution of each word to the final prediction. We implemented this model in OpenNRE under the MIL setting.

BERE [6]: the model adopts a hybrid encoding network to represent each sentence from both semantic and syntactic aspects. First, the model encodes each sentence through a transformer layer [8] to capture long-range dependencies. Secondly, it uses a BiGRU to capture local contextual features and then applies a Gumbel Tree-GRU [9] to organize words into nested phrases. Finally, the model concatenates contextual features of target entities with sentence features obtained by Gumbel Tree-GRU to derive entity-aware, sentence-level features. We implemented this model in OpenNRE under the MIL setting.

All models use pre-trained word embeddings to initialize word representations. On the other hand, PFs, PIs, and unknown words are initialized using the normal distribution, whereas blank words are initialized with zeros.

Parameter settings

We adopted pre-trained BioWordVec [10] embeddings to perform experiments on TBGA. BioWordVec combines subword information obtained from biomedical literature with knowledge from Medical Subject Headings (MeSH). Two versions of pre-trained BioWordVec embeddings are available: "Bio_embedding_intrinsic" and "Bio_embedding_extrinsic". The former can be used to compute semantic similarity between words, terms, or sentences. The latter can be used as input for several downstream applications, such as relation extraction or text classification. Both versions contain 2,324,849 distinct words and have an embedding size of 200. BioWordVec embeddings are publicly available on Figshare [11]. We chose the "Bio_embedding_extrinsic" version as it is the most suitable for Biomedical Relation Extraction (BioRE). As for the experiments on DTI and BioRel, we adopted the pre-trained word embeddings used in the original works [6, 5] – that is, the word embeddings from Pyysalo et al. [12] for DTI, and the "Bio_embedding_extrinsic" version of BioWordVec for BioRel.

For TBGA experiments, we used grid search to determine the best combination between optimizer and learning rate. As combinations, we tested Stochastic Gradi-

Table S1: The hyper-parameter settings of the RE models for TBGA. The best combination between optimizer and learning rate was obtained through grid search, independently for each aggregation strategy considered: average-based (AVE) and attention-based (ATT). The tested combinations were SGD with learning rate among {0.1, 0.2, 0.3, 0.4, 0.5} and Adam [13] with learning rate set to 0.0001. The rest of the hyper-parameters were set empirically. The "–" symbol means that the considered hyper-parameter does not apply to the specific RE model.

Hyper-parameters	CNN	PCNN	BiGRU	BiGRU-ATT	BERE
Word dim	200	200	200	200	200
Position dim	10	10	–	–	–
Part-of-speech dim	–	–	–	–	50
Sentence dim	250	250	250	250	250
Learning rate (AVE)	0.2	0.1	0.5	0.5	0.0001
Learning rate (ATT)	0.0001	0.1	0.4	0.2	0.2
Dropout	0.5	0.5	0.5	0.5	0.5
Batch size	64	64	64	64	64
Maximum epoch	20	20	20	20	20

ent Descent (SGD) with learning rate among {0.1, 0.2, 0.3, 0.4, 0.5} and Adam [13] with learning rate set to 0.0001. For all RE models, we set the rest of the hyper-parameters empirically. Table S1 reports the optimal hyper-parameter settings.

For DTI and BioRel experiments, we relied on the hyper-parameter settings reported in the original works [6, 5].

Abbreviations

BiGRU Bidirectional Gated Recurrent Unit Neural Network
BioRE Biomedical Relation Extraction
CNN Convolutional Neural Network
MIL Multi-Instance Learning
MeSH Medical Subject Headings
PCNN Piecewise Convolutional Neural Network
PF Position Feature
PI Position Indicator
RE Relation Extraction
SGD Stochastic Gradient Descent

Author details

Department of Information Engineering, University of Padova, Padova, Italy.

References

1. Zeng D, Liu K, Lai S, Zhou G, Zhao J. Relation Classification via Convolutional Deep Neural Network. In: Proc. of COLING 2014, 25th International Conference on Computational Linguistics, Technical Papers, August 23-29, 2014, Dublin, Ireland. ACL; 2014. p. 2335–2344.

2. Han X, Gao T, Yao Y, Ye D, Liu Z, Sun M. OpenNRE: An Open and Extensible Toolkit for Neural Relation Extraction. In: Proc. of the 2019 Conference on Empirical Methods in Natural Language Processing and the 9th International Joint Conference on Natural Language Processing, EMNLP-IJCNLP 2019, Hong Kong, China, November 3-7, 2019. ACL; 2019. p. 169–174.
3. Zeng D, Liu K, Chen Y, Zhao J. Distant Supervision for Relation Extraction via Piecewise Convolutional Neural Networks. In: Proc. of the 2015 Conference on Empirical Methods in Natural Language Processing, EMNLP 2015, Lisbon, Portugal, September 17-21, 2015. ACL; 2015. p. 1753–1762.
4. Zhang D, Wang D. Relation Classification via Recurrent Neural Network. CoRR. 2015;abs/1508.01006.
5. Xing R, Luo J, Song T. BioRel: towards large-scale biomedical relation extraction. BMC Bioinform. 2020;21-S(16):543.
6. Hong L, Lin J, Li S, Wan F, Yang H, Jiang T, et al. A novel machine learning framework for automated biomedical relation extraction from large-scale literature repositories. Nat Mach Intell. 2020;2:347–355.
7. Zhou P, Shi W, Tian J, Qi Z, Li B, Hao H, et al. Attention-Based Bidirectional Long Short-Term Memory Networks for Relation Classification. In: Proc. of the 54th Annual Meeting of the Association for Computational Linguistics, ACL 2016, August 7-12, 2016, Berlin, Germany, Volume 2: Short Papers. ACL; 2016. p. 207–212.
8. Vaswani A, Shazeer N, Parmar N, Uszkoreit J, Jones L, Gomez AN, et al. Attention is All you Need. In: Adv. in Neural Information Processing Systems 30: Annual Conference on Neural Information Processing Systems 2017, December 4-9, 2017, Long Beach, CA, USA; 2017. p. 5998–6008.
9. Choi J, Yoo KM, Lee SG. Learning to Compose Task-Specific Tree Structures. In: Proc. of the Thirty-Second AAAI Conference on Artificial Intelligence, (AAAI-18), the 30th innovative Applications of Artificial Intelligence (IAAI-18), and the 8th AAAI Symposium on Educational Advances in Artificial Intelligence (EAAI-18), New Orleans, Louisiana, USA, February 2-7, 2018. AAAI Press; 2018. p. 5094–5101.
10. Zhang Y, Chen Q, Yang Z, Lin H, Lu Z. BioWordVec, improving biomedical word embeddings with subword information and MeSH. Sci Data. 2019;6(52):1–9.
11. Zhang Y, Chen Q, Yang Z, Lin H, Lu Z. BioWordVec: Improving Biomedical Word Embeddings with Subword Information and MeSH Ontology. Figshare; 2018. Available from: https://figshare.com/articles/dataset/Improving_Biomedical_Word_Embeddings_with_Subword_Information_and_MeSH_Ontology/6882647/2.
12. Pyysalo S, Ginter F, Moen H, Salakoski T, Ananiadou S. Distributional Semantics Resources for Biomedical Text Processing. Proc of LBM. 2013;p. 39–44.
13. Kingma DP, Ba J. Adam: A Method for Stochastic Optimization. In: Proc. of the 3rd International Conference on Learning Representations, ICLR 2015, San Diego, CA, USA, May 7-9, 2015; 2015. p. 1–15.
