# Supplementary material for: TBGA: a large-scale Gene-Disease Association dataset for Biomedical Relation Extraction
Source: BMC Bioinformatics. 2022 Mar 31;23:111. doi: 10.1186/s12859-022-04646-6 (PMC8973894; doi:10.1186/s12859-022-04646-6)
Supplement: Supplementary file 2 — Additional file 2. Baselines validation. Results and discussion of the experiment performed to validate the soundness of the implementation of theconsidered RE models. [file 12859_2022_4646_MOESM2_ESM.pdf]

ADDITIONAL FILE 2

TBGA: A Large-Scale Gene-Disease Association Dataset for Biomedical Relation Extraction

Stefano Marchesin* and Gianmaria Silvello

*Correspondence: stefano.marchesin@unipd.it
Department of Information Engineering, University of Padova, Padova, Italy
Full list of author information is available at the end of the article

Experimental results

Baselines validation

The results of the baselines validation are reported in Table S2. We can observe that the Relation Extraction (RE) models we used from – or implemented within – OpenNRE achieve performance higher than or comparable to those reported in DTI and BioRel original works. The only exceptions are BiGRU and BiGRU-ATT on DTI, where the Area Under the Precision-Recall Curve (AUPRC) scores of our implementations are lower than those reported in the original work. However, Hong et al. [1] report the optimal hyper-parameter settings for BERE, but not for the baselines. Thus, we attribute the negative difference between our implementations and theirs to the lack of information about optimal hyper-parameters. Overall, the results confirm the soundness of our implementations. Therefore, we can consider them as competitive baseline models to use for benchmarking Gene-Disease Association (GDA) extraction.

Abbreviations

AUPRC Area Under the Precision-Recall Curve
GDA Gene-Disease Association
RE Relation Extraction

Author details

Department of Information Engineering, University of Padova, Padova, Italy.

References

1. Hong L, Lin J, Li S, Wan F, Yang H, Jiang T, et al. A novel machine learning framework for automated biomedical relation extraction from large-scale literature repositories. Nat Mach Intell. 2020;2:347–355.
2. Xing R, Luo J, Song T. BioRel: towards large-scale biomedical relation extraction. BMC Bioinform. 2020;21-S(16):543.

Table S2: Results of the baselines validation on DTI [1] and BioRel [2] datasets. Columns represent, from left to right, the considered RE model, the aggregation strategy, the reproduced or reference implementation, and the AUPRC scores for DTI and BioRel, respectively. The "–" symbol means that the RE model, for the given aggregation strategy, has not been originally evaluated on the specific dataset.

Model	Strategy	Implementation	DTI	BioRel
CNN	AVE	Reproduced	–	0.800
		Original	–	0.790
	ATT	Reproduced	–	0.790
		Original	–	0.780
PCNN	AVE	Reproduced	0.234	0.860
		Original	0.160	0.820
	ATT	Reproduced	0.408	0.820
		Original	0.359	0.790
BiGRU	AVE	Reproduced	–	0.870
		Original	–	0.800
	ATT	Reproduced	0.379	0.850
		Original	0.390	0.780
BiGRU-ATT	ATT	Reproduced	0.383	–
		Original	0.457	–
BERE	AVE	Reproduced	0.407	–
		Original	0.384	–
	ATT	Reproduced	0.525	–
		Original	0.524	–
